# Dimensions of Social Isolation and Weight Loss among Older Men and Women in S. Korea

**DOI:** 10.1093/geroni/igaa057.3343

**Published:** 2020-12-16

**Authors:** Youngjoon Bae, Mark Pachucki

**Affiliations:** University of Massachusetts Amherst, Amherst, Massachusetts, United States

## Abstract

Older men who live alone are typically believed to be highly susceptible to malnutrition. However, recent studies report their living alone status is associated with frailty negatively and with Type 2 diabetes positively. Meanwhile, older women who live alone are believed to be less susceptible to malnutrition, but qualitative research point out their high likelihood of malnutrition. There is little literature to explain these paradoxes. To evaluate this gap in understanding of how a metabolic process may be shaped by social context, this study examines whether different aspects of social isolation among older men and women (living alone, social contact, loneliness) are associated with adverse weight loss, as well as with indicators of meal frequency and body weight. For this, a data set comprised of 6,680 older adults from the Korean Longitudinal Study of Aging surveyed every two years from 2006 to 2018 was analyzed using panel regression models. Among older men, living alone was negatively associated with logged body weight even when considering loneliness but not associated with meal frequency and 5kg or more weight loss. Among older women, living alone was not associated with logged body weight but associated with fewer meals and 5kg or more weight loss. The association disappeared when considering loneliness. Unexpectedly, social contact was not significant to prevent adverse weight loss.

